# Clocks on steroids: how glucocorticoid receptors tell cells the time

**DOI:** 10.1530/JOE-25-0340

**Published:** 2026-04-08

**Authors:** Anna Edmondson, Joseph Menzies, David W Ray, John S O’Neill

**Affiliations:** ^1^ MRC Laboratory of Molecular Biology, Cambridge, UK; ^2^ Oxford Centre for Diabetes, Endocrinology and Metabolism, Oxford, UK

**Keywords:** Glucocorticoid receptor, Cell biology, Circadian rhythms, Glucocorticoid

## Abstract

Glucocorticoid (GC) steroid hormone signalling via the glucocorticoid receptor (GR) underlies most daily physiological rhythms in mammals by synchronising cellular circadian rhythms throughout the body. Impaired circadian synchrony is associated with many non-communicable diseases, such as cardiovascular disease, diabetes and cancer. The functions and daily regulation of systemic GC levels are relatively well understood, yet a clearly defined mechanism for GC/GR-mediated circadian synchronisation is lacking. Historically, mechanistic studies of GR action have focused on its role as a transcription factor, sufficient to explain many, but not all, consequences of GC/GR signalling. Recently, several non-canonical modes of GR action have been described and proposed as a basis for understanding rapid cellular responses to GCs that cannot be explained by relatively slow changes in transcription. Here, we review the current state of knowledge on the cellular mechanism of GR signalling in the context of GR-mediated circadian synchronisation, outline gaps in current understanding and suggest new avenues for investigation.

## Introduction

Circadian rhythms are the ∼24 h endogenous cycles in behaviour, physiology, metabolism and cellular activity exhibited by most organisms and are readily observed in cultured cells and tissues *ex vivo*, persisting under constant conditions over many days-weeks. Cellular circadian rhythms are driven by an intrinsic ∼24 h timing mechanism (henceforth termed cellular clock), which can function autonomously. *In vivo*, however, daily rhythms in gene expression, protein activity and cellular function result from the interaction between cellular clocks and centrally coordinated systemic timing cues, such as glucocorticoid (GC) signalling. Systemic cues, such as GC signalling, change in response to external factors, such as light exposure (1), synchronising cellular clocks throughout the body with the 24 h rhythm of day and night.

**Figure 1:**
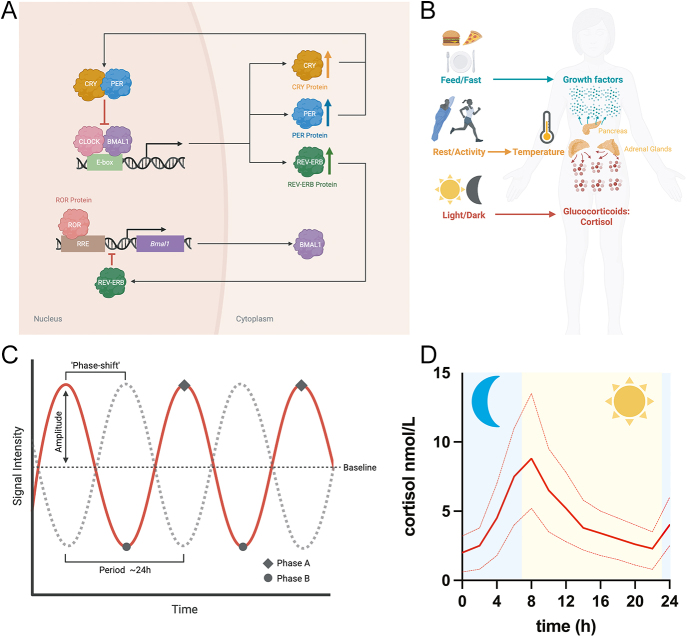
Transcription–translation feedback loop (TTFL) model, circadian oscillations and physiological circadian synchronisation. (A) In the simplest iteration of the TTFL model, CLOCK and BMAL1 drive PERIOD (*PER*), CRYPTOCHROME (*CRY*) and *NR1D1* transcription. PER/CRY complexes feedback to inhibit CLOCK/BMAL1, and auxiliary loops (e.g. REV-ERB/ROR) reinforce BMAL1 oscillations (2) due to rhythmic REV-ERBa/B expression. RRE, ROR response element; E-box, enhancer box. (B) There are three major external synchronising cues (zeitgebers) in mammals: i) feed/fast cycles, ii) rest/activity cycles and iii) light/dark cycles. Feed/fast cycles are communicated by growth factor signalling (3), body temperature changes correlate with rest/activity cycles (4), whereas GCs signal environmental light/dark cycles. (C) Circadian oscillations can be represented as a sine wave (red line). Period describes the duration of each oscillation (∼24 h). Amplitude describes the distance between the baseline (black dotted line) and y-axis peak, or trough. Phase describes a discrete stage within the oscillation, e.g. the peak (phase A) or trough (phase B). A phase shift refers to when an oscillation is translated in time along the x-axis (red to grey dotted line). (D) Daily fluctuation in the human interstitial fluid concentration of cortisol, *n* = 214. The solid red line represents mean cortisol concentration; the dashed lines represent 25% and 75% quartiles, adapted from Upton *et al.* (5). A full colour version of this figure is available at https://doi.org/10.1530/JOE-25-0340.

Circadian rhythms can be represented as a sine wave, where a single peak and trough occurs within each ∼24 h period. The term ‘phase’ describes the point at which the oscillation resides at a given moment in time. When cells receive a synchronising cue at the ‘wrong’ physiological time, e.g. 12 h before or after anticipated, it causes the phase of oscillation to shift, i.e. the sine wave is translated in time along the x-axis (Fig. 1C). For the purposes of this review, the effect that synchronising cues have on circadian oscillations will be described as ‘phase-resetting’.

In mammals, circadian phase is determined by several external synchronising cues (zeitgebers) that normally vary over 24 h due to changes in behaviour and the environment, i.e. light/dark cycles, fed/fast cycles (3) and rest/activity cycles (4). External cues are relayed to cells throughout the body via internal messengers (Fig. 1B). Whilst GC signalling is affected by stress, feed/fast and rest/activity, physiologically, rhythmic GC signalling represents the integration of actual and anticipated daily variation in light exposure. This is coordinated by a central clock, the suprachiasmatic nucleus (SCN), via the hypothalamic–pituitary–adrenal (HPA) axis (6). In diurnal humans, HPA activity coordinates the production and release of cortisol from the adrenal glands a few hours before dawn (or habitual wake time) (7). In nocturnal mice, corticosterone levels are highest a few hours before (anticipated) dusk (8). GCs readily diffuse into cells, where they act as a potent synchronising cue (9). Moreover, in the absence of the SCN and adrenal glands, 24 h rhythms in exogenously applied GCs are reported to drive rhythms of clock gene expression in non-SCN brain regions (10). In this way, at the organismal, tissue and cellular level, GC signalling maintains the robust and synchronised oscillations required for optimal physiological function and cellular homoeostasis (6).

The mammalian cellular clock is facilitated by a transcription–translation feedback loop (TTFL). Briefly, the transcriptional activators, circadian locomotor output cycles kaput (CLOCK) and basic helix–loop–helix ARNT-like 1 (BMAL1) heterodimerises and binds upstream of the PERIOD (*PER*) and CRYPTOCHROME (*CRY*) genes, promoting their transcription. This leads to a subsequent increase in the abundance of the encoded PER and CRY proteins, which form a complex that feeds back to inhibit the activity of CLOCK and BMAL1, thereby repressing *PER* and *CRY* transcription. The resultant decrease in PER and CRY proteins releases the inhibition on CLOCK and BMAL1, closing the loop (Fig. 1A). Canonically, the ∼24 h taken to complete this circuit dictates the periodicity of downstream oscillations (11). In this way, the TTFL is thought to drive downstream gene expression programmes that generate daily rhythms in organismal physiology via ‘clock-controlled genes’ (11).

Further work has revealed greater complexity to the TTFL model, expanding it to encompass additional interconnected transcriptional, translational and post-translational feedback loops (12). However, whilst the TTFL occurs in most mammalian cell types and many of its components are required for circadian function, the direct evidence that the TTFL determines circadian periodicity or physiology is weak (discussed in (13, 14)). Yet, the evidence that PER protein activity is an essential clock component is extremely strong (15, 16).

GC steroid hormone signalling via the glucocorticoid receptor (GR) is important for synchronising circadian rhythms in most mammalian cell types (17). Transcriptomics of mouse liver has indicated that GC signalling determines the timing of most circadian gene expression (18), and also identified >100 genes, including those of metabolic enzymes, whose daily expression rhythm is GC-dependent (19). However, whilst most hepatic transcripts that oscillate in abundance are directly regulated by the GR (20), there is some redundancy with regulation due to daily cycles of feed/fast and insulin signalling. Indeed, hepatocyte-specific GR knockout (KO) only attenuates most daily gene expression rhythms rather than abolishing them (21).

GC signalling coordinates diverse physiological systems (22), and synthetic GCs’ broad application within medicine has therefore stimulated in-depth studies of the GR’s cellular signalling mechanisms, and role as a transcription factor (TF), which is sufficient to explain many, but not all biological effects elicited by GCs. How GCs synchronise cellular clocks is not completely understood, and currently, no resource describes a detailed cellular mechanism. Here, we review the literature on cellular GR signalling, focusing on the mechanism by which GCs synchronise cellular circadian rhythms. We explore developments in non-canonical GR signalling and highlight areas where research into the GR-to-clock signalling mechanism is needed. Since circadian regulatory mechanisms and GC/GR signalling are highly conserved amongst mammals, we refer to the human form of genes/proteins throughout this review.

## Structure of the GR

Encoded by the nuclear receptor subfamily 3 group C member 1 (*NR3C1*) gene, the GR is a member of the 3-ketosteroid receptor subfamily of TFs (23). Members of this subfamily share a conserved modular structure comprising four domains: the ligand-binding domain (LBD), the hinge, the DNA-binding domain (DBD) and the N-terminal domain (NTD). The sequence of the GR is highly evolutionarily conserved amongst mammals, and the functionality of mammalian GRs are identical (24).

The LBD contains a hydrophobic ligand binding pocket that is structurally similar within nuclear receptor subfamily 3, resulting in partial cross-reactivity between receptors and ligands. GCs have a higher affinity for the mineralocorticoid receptor (MR) than the GR (25), meaning that the MR is largely GC-saturated *in vivo*. Thus, the GR likely mediates the daily dynamics of GC signalling (25). However, evidence supporting exclusive dependence on the GR for GC-resetting is largely based on the observation that the GR antagonist, mifepristone (MIF) abolishes DEX-mediated phase shifts (26, 27). As MIF also potently inhibits the progesterone receptor (PR) (28), it remains uncertain whether GC-synchronisation acts solely through the GR.

### Post-translational modifications (PTMs) of the GR

The GR can undergo numerous PTMs, including phosphorylation, sumoylation, acetylation, ubiquitylation and nitrosylation (29). Proteomics has identified over 80 PTM sites (30), although it is sumoylation and acetylation of the GR that have been associated with the clock.

Sumoylation is associated with GR degradation and exhibits both ultradian and circadian rhythmicity, reflecting pulsatile GC release from the adrenal glands (31). Prolonged GC treatment disrupts rhythmic GR sumoylation, suggesting that this functions to maintain GR sensitivity.

Acetylation sites within the hinge region at Lys480, Lys492, Lys494 and Lys495 have been proposed to negatively regulate the GR’s transcriptional activity (32). Nader *et al.* reported that this is mediated by CLOCK/BMAL1 and is dependent on CLOCK’s (HAT) activity. Furthermore, knockdown of CLOCK and BMAL1 was found to enhance GR-mediated transcriptional activation, whilst CLOCK/BMAL1 overexpression was reported to repress transcriptional activation and enhance acetylation at these sites. Interestingly, the GR was shown to interact with CLOCK via its LBD independently of its acetylation, an interaction that requires GC ligand. Given the rhythmic nature of CLOCK/BMAL1 activity, the authors speculate that GR acetylation occurs in a circadian manner.

However, acetylation was only clearly observed in an overexpression context. Moreover, mutation of acetylated lysine residues has pleiotropic effects on the central NLS1 motif, rendering the GR constitutively cytoplasmic. Therefore, reduced transcriptional activity of GR ‘acetylation’ mutants upon GC addition likely reflects impaired nuclear localisation, not a reduction in the GR’s DNA-binding affinity as proposed.

## Classical transcriptional signalling pathway and circadian interactions

**Figure 2:**
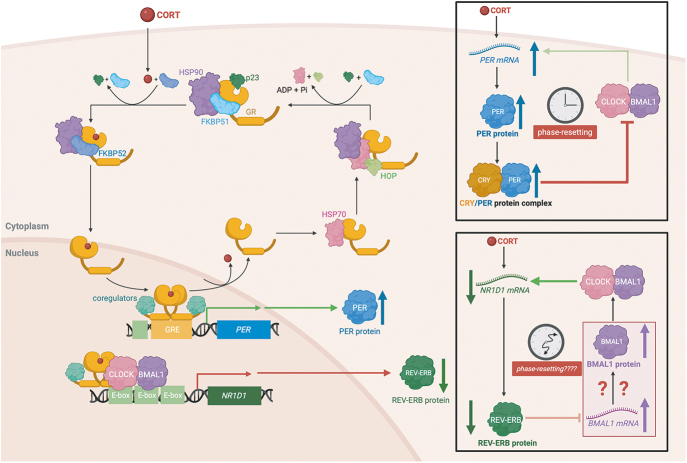
Classical cellular model of GR signalling and circadian phase-resetting by GCs. In the absence of ligand, the GR resides in a cytoplasmic complex comprised of HSP90, p23 and FKBP51. Ligand-binding to the GR induces a conformational change in the receptor that causes FKBP51 to be replaced by FKBP52 and facilitates nuclear translocation of the GR. In the nucleus, the GR binds to GREs in proximity of its target genes, activating or repressing their transcription through co-activator or co-repressor recruitment. In GC-synchronisation, the GR binds to GREs upstream of the *PER* genes, leading to increased *PER* mRNA and PER protein abundance. The change in PER abundance shifts the phase of the cellular clock (15) through increased inhibition of CLOCK/BMAL1, regardless of the pre-existing phase of the TTFL (top black box). The GR also binds at E-boxes upstream of *NR1D1* through tethering to CLOCK/BMAL1, leading to *NR1D1* repression, and a decrease in the abundance of REV-ERBa. Ligand-bound GR is recycled in the cytoplasm through the activity of HSP70 and HOP. CORT, cortisol; FKBP5, FK506-binding protein 5; GRE, glucocorticoid response element; *PER*, Period; HSP, heat shock protein; HOP, Hsp70–Hsp90 organising protein; AF, activation function. A full colour version of this figure is available at https://doi.org/10.1530/JOE-25-0340.

PER protein activity is the central determinant of mammalian cellular circadian phase (15). Essentially all mechanisms of phase-resetting are ultimately thought to occur through changing PER protein activity by regulating *PER* transcription via promoter response elements. Appealingly, the textbook model of GC signalling via the GR centres on its function as a TF, where GCs bind to and activate monomeric GR within the cytoplasm by releasing it from an inactive chaperone-bound state, thereby stimulating its nuclear translocation. Once nuclear-localised, the GR homodimerises upon binding to glucocorticoid response elements (GREs): specific DNA sequences upstream of the GR’s target genes, regulating their transcription through the recruitment of co-activator or co-repressor complexes. Resulting changes in the cellular transcriptome are postulated to drive the observed biological effects of GC signalling (Fig. 2).

Similarly, to synchronise cells, GC-bound GR binds to GREs upstream of the *PERIOD* genes, promoting their transcription, leading to increased abundance of *PER* mRNAs and the encoded PER proteins. CRY protein copy number exceeds that of PER, so it is PER abundance that is limiting for CRY/PER complex formation, and hence the driving force for circadian phase-resetting, as the concordant increase in CLOCK/BMAL1 inhibition would occur regardless of the antecedent phase of the core TTFL circuit (Fig. 2). There are three closely related paralogues of PER, but only PER1 and PER2 are necessary for the maintenance and synchronisation of circadian oscillations. GC-bound GR also binds upstream and represses transcription of *NR1D1*, which encodes the auxiliary clock protein REV-ERBa. The decrease in REV-ERBa abundance releases inhibition on *BMAL1* transcription. Induction of *BMAL1* transcription would plausibly elicit circadian phase-resetting if the availability of BMAL1 were rate-limiting for TTFL activity at any phase of the circadian cycle. However, this has yet to be demonstrated.

### Nucleocytoplasmic trafficking and GR recycling

Prior to ligand binding, the GR monomer is thought to reside in an inactive cytoplasmic complex. Upon ligand-binding, conformational changes drive nuclear translocation of the GR (33). Live-cell imaging studies have demonstrated that following ligand-binding, the onset of GR nuclear translocation occurs within 5 min but only reaches saturation after 30 min (34). On the contrary, changes in nascent transcription are observed within 10 min of GC treatment (35).

The half-life of the liganded GR is of the order of minutes (36). In the classical model, GC dissociation occurs spontaneously and unliganded GR is exported to the cytoplasm where it is recycled (37). Recycling of the GR is less energetically costly than degrading and replacing the activated protein but still allows for signalling with a high signal-to-noise ratio.

GR nucleocytoplasmic localisation follows a daily rhythm in mouse liver *in vivo*, peaking early in the active phase (ZT12-15) immediately following the habitual increase in plasma GCs around subjective dusk and preceding the nuclear accumulation of REV-ERBa/B by 3–6 h (8, 38, 39). Diurnal subcellular localisation of the GR appears to be indirectly regulated by REV-ERBa as REV-ERBa-deficient mice were shown to have increased nuclear GR in both the day and night.

### Transcriptional regulation

The GR activates or represses of an array of genes through several distinct DNA-binding modes (35, 40). In the context of GC-resetting, the GR has been reported to activate the *PER1* and *PER2* genes and repress *NR1D1* (encoding REV-ERBa). The GR achieves its transcriptional activities by localising large transcriptional complexes to discrete genomic loci termed glucocorticoid response elements (GREs), making both specific DNA and context-dependent co-regulator interactions.

Central to the GC-synchronisation model is the observation that dexamethasone (DEX), a synthetic GC, acutely induces *PER1*, but not *PER2*, mRNA expression in rat fibroblasts and drives sustained oscillations in other clock genes/proteins (17). Using mice harbouring liver-specific disruption of the GR, the authors demonstrated that the induction of *PER1* mRNA depends on a functional GR. This initial mechanistic work suggested *PER1*, not *PER2* to be the primary mediator of GC-synchronisation. Analogous results have been obtained by RT-qPCR across multiple tissues, in rats injected with GCs, and by RNA-seq in human cells, demonstrating the physiological relevance and mammalian conservation of rapid *PER1* induction (41, 42, 43). Genome-wide, *PER1* exhibited the highest magnitude of fold-change in mRNA expression 1 h after treatment with low doses (0.1–5 nM) of DEX (43). Importantly, Reddy *et al.* revealed an accompanying increase in PER1 protein, 4 h following DEX addition.

Later studies have suggested that *PER2* transcription is also acutely induced by DEX. Although induction of *PER2* is more sustained than *PER1*, it possesses slower kinetics and is of a much lower magnitude (26, 27, 41). Moreover, *in vivo* the peak of *PER1* mRNA expression occurs concurrently with the peak in plasma corticosterone concentration in adipose, liver and jejunum tissue, whereas *PER2* mRNA peaks ∼4 h later (19).

Which *PER*/PER paralogue is important for phase-resetting may differ depending on the tissue type (44). Removal of plasma corticosterone by adrenalectomy only abolished *PER1* rhythms in splenocytes, adipose, liver and jejunum tissue but not the kidney, where it was *PER2* rhythms that were severely attenuated. Conversely, other studies report that *PER* responses to GCs are similar across multiple different cell and tissue types (17, 42).

On the other hand, another circadian gene, *NR1D1*, is robustly downregulated following the addition of GCs (45, 46). Using MIF and actinomycin B (a transcriptional inhibitor), Torra *et al.* reported that *NR1D1* downregulation is mediated by the GR transcriptionally. Furthermore, Murayama *et al.* showed that *NR1D1* expression increases following adrenalectomy. Interestingly, the authors demonstrated that *PER1* knockdown did not affect *NR1D1* downregulation by GCs.

*In vivo*, synthetic GCs can shift the phase of circadian-regulated genes in peripheral tissues, but not in the adult SCN (17). Although the SCN was initially reported not to express *NR3C1* (GR) mRNA in the adult (17), more recent work has found the *GR* mRNA and functional GR transcriptional signalling in adult SCN. However, unlike most other tissue and cell types, GCs do not affect circadian timing in adult SCN. The remarkable resilience of SCN rhythms to perturbation has also been demonstrated for other hormonal timing cues, such as insulin, and arises through the unique nature of this brain structure’s intercellular neuropeptidergic coupling (3, 47, 48).

### Modes of DNA binding

The classical mode of GR-mediated transcriptional activation involves canonical GRE sequences, which are two pseudo-palindromic hexameric repeats separated by three nucleotides, the consensus being AGAACA-(nnn)-TGTTCT. Dimeric GR binds to these sequences with high affinity in a head-to-head fashion (29) to nucleate the formation of relatively stable transcriptional activation complexes. However, amongst functional GREs, there is significant sequence degeneracy. GRE binding by the GR is also influenced by chromatin accessibility at these loci, which is affected in turn by the presence of other proximal TFs (49). The GR can also bind to non-canonical GREs and functions in several different oligomerisation states that vary with GRE context (40, 50, 51, 52, 53).

### Interactions with circadian genes and clock proteins

In a circadian context, a highly conserved distal GRE sequence has been identified through *in silico* analysis upstream of *PER1*, only differing from the canonical consensus GRE by a single base (AGA ACA nnn TGT TCC) (42). Yamamoto *et al.* demonstrated by ChIP-seq that this GRE is GR-bound and required for DEX-mediated *PER1* induction. In addition, Reddy *et al.* identified an enhancer region upstream of the *PER1* transcription start site (TSS), which they speculate contains additional regulatory sequences that amplify *PER1* transcriptional activation at low GC doses (43).

Evolutionarily conserved GREs occupied by the GR upon DEX treatment have also been identified upstream of *PER2* (26, 27). However, *PER2* GREs showed a lower sensitivity than *PER1* GREs, with GR occupancy at these *PER2* sites being <15% that of *PER1* sites under the same conditions (26). Importantly, cell-autonomous rhythms in *PER2* mRNA expression cannot be mediated by the GR; GR occupancy of the putative *PER2* GRE does not significantly change over 48 h in mouse mesenchymal stem cells (MSCs). Deletion of a promoter region containing this sequence in mice *in vivo* abolished DEX-induced oscillations in *PER2* transcripts and damped cycling of other clock transcripts (26). However, there is poor agreement on the identity of the specific GRE responsible for *PER2* induction (26, 27). Moreover, in addition to the *PER2* GRE, an E-box upstream of *PER2* is reportedly required for its induction (27).

By contrast, transcription from the *NR1D1* promoter is repressed following DEX addition (45), with ChIP-seq data revealing that the GR binds within the *NR1D1* promoter region. Murayama *et al.* also showed by ChIP that the GR interacts with three E-boxes upstream of *NR1D1* in a DEX-dependent manner. Intriguingly, GR-mediated repression of *NR1D1* promoter activity apparently occurs in the absence of direct GR-DNA binding and is dependent on the presence of CLOCK and BMAL1 (45). The authors speculate that repression of REV-ERBa is achieved via the GR ‘tethering’ to the CLOCK/BMAL1 complex situated on the E-boxes upstream of *NR1D1* (45). In this way, Murayama *et al.* proposed that it is the CLOCK/BMAL1-dependent repression of REV-ERBa that causes GC-synchronisation. In support, CLOCK has been demonstrated to interact directly with the GR both *in vivo* and *in vitro* (32, 45).

There is evidence that GR binding at ‘functionally relevant’ *PER2* GREs depends on its association with BMAL1 (27) and that the presence of BMAL1 is essential for *PER2* induction. As such, both BMAL1 and CLOCK are considered important for GC-synchronisation, with direct interactions with the GR deemed essential for activation of *PER2* and repression of *NR1D1*, respectively. However, BMAL1 and CLOCK are required for basal expression of *PER2* and *NR1D1* and may simply be permissive for GR-mediated changes in transcription by maintaining local chromatin structure rather than playing an active role in resetting.

Other clock-associated GR co-regulators have also been identified. CRY proteins have been shown to interact with ligand-bound GR (54, 55), acting to oppose transcriptional activation of the GR’s target genes on chromatin (54). RNA microarray analysis following DEX stimulation of *CRY1*^*−/−*^*CRY2*^*−/−*^ double KO (CRY DKO) mice revealed that CRY absence leads to a greater transcriptional network response and higher magnitude of *PER1* induction than wild-type (WT) mice (54). The authors propose that repression of the GR by CRY limits the induction of a larger network of GR-responsive genes. Most interestingly, CRY DKO mice exhibited constitutively high levels of circulating corticosterone, meaning that GC-mediated cues to the cellular clock are absent without CRY and likely contribute to the pleiotropic phenotype of these animals. Overall, the evidence suggests that CRY proteins are permissive for and may modulate GC cues to the cellular clockwork but are not directly involved in phase-resetting.

Similarly, REV-ERBa has been reported to selectively regulate GR function in a phase-dependent manner, independently of CRY (55). Caratti *et al.* used ChIP-seq to show that REV-ERBa and the GR bind to the same genomic regions within mouse liver and reported a direct physical interaction between REV-ERBa and the GR regardless of the presence of GC ligand. Although REV-ERBa KO mice indicate that REV-ERBa does not influence the anti-inflammatory effects of GCs, the temporal characteristics of the GC response are significantly perturbed its absence (55).

### Biomolecular condensates

Recent discoveries have suggested that the GR participates in higher-order heterogeneous macromolecular assemblies referred to as biomolecular condensates (BMCs), which are enriched in co-regulator proteins (56). BMCs are characterised by multivalent interactions between RNA and intrinsically disordered regions of proteins. Transcriptional BMCs therefore concentrate TFs, transcriptional co-regulators and RNA polymerase II at discrete genomic loci (57). As the N-terminal half of the GR is intrinsically disordered, its participation in BMCs is quite plausible and is speculated to enhance its transcriptional regulatory capacity. In solution, the GR has been shown to form such homo- and heteromeric condensates with other TFs (58). Furthermore, single-molecule tracking studies within the nuclear compartment have suggested that the activated GR exhibits two distinct sub-diffusive low-mobility states, i.e. modes of genomic association: one mediated by specific chromatin interactions, whilst the other a more dynamic state dependent on the IDR (56).

Given that the GR’s proposed circadian transcriptional co-regulators, CLOCK, BMAL1, CRY and REV-ERBa, all contain IDRs (59), it is plausible that they also participate in GR transcriptional BMCs.

## Non-canonical pathways

Whilst the model of GR signalling described above is supported by many observations, recent research has revealed further complexity surrounding the GR’s diverse transcriptional and entirely non-transcriptional activities (34). For example, activation of the GR results in rapid effects (seconds to minutes) on immunosuppression and the vascular system that cannot be explained by relatively slow changes in the transcriptome (60, 61). It is therefore important to consider the various non-canonical modes by which GRs have been proposed to act in the context of GC-synchronisation: 1) via membrane-bound GR and caveolin-1 (CAV1), 2) via cytoplasmic kinases such as Src and phosphoinositide 3-kinase (PI3K) and 3) via direct regulation of mRNA (Table 1).

**Table 1:** Summary of reported non-transcriptional GR actions.

	Key research papers	Physiological context	Relevance to clock regulation
Non-transcriptional GR action			
Membrane-bound GR and caveolin-1 (CAV-1)	(62, 63, 64, 65, 66, 67, 68)	Cell proliferation Apoptosis Immune modulation Metabolism Interaction with reproductive signalling	Modulates GC phase shifts Increases the kinetics of clock gene mRNA upregulation
Cytoplasmic kinase signalling			
Src	(60, 63, 64, 69)	Anti-inflammatory signalling Cell (neuronal) proliferation Apoptosis Activation of eNOS in the vascular system	Feedback mechanism: inhibition of ACTH secretion Potential contributor to GC-synchronisation
PI3K	(70, 71, 72, 73)		
MAPK	(64, 69, 71, 74)		
PKA and MEK	(75)		
mRNA regulation			
GR-mediated mRNA decay	(76, 77, 78, 79)	Anti-inflammatory response	*PER1* and *PER2* as putative GR mRNA binding targets (based on *in silico* data)
RNA binding via hairpin loop motif			
Translational regulation and protein turnover	(75, 80)	Anti-inflammatory response	PER2 *de novo* protein synthesis Modulation of PER2 protein turnover

### Membrane GR and CAV-1

GC-mediated kinase signalling cascades are largely attributed to membrane GR. Disruption of lipid raft signalling in mouse lung slices and Rat-1 cells via methyl cyclodextrin administration has been reported to reduce the magnitude of CORT-mediated phase shifts (81). Interestingly, cells derived from CAV-1^−/−^ mice exhibited CORT-mediated *PER2* inductions with significantly slower kinetics than WT cells (81). These findings suggest that membrane GR activity influences GC-synchronisation by modulating PER2 induction.

### Cytoplasmic kinases

Rapid consequences of GR activation reportedly involve cytoplasmic serine/threonine kinases, such as mitogen-activated protein kinase (MAPK), phosphoinositide 3-kinases (PI3K) and protein kinase C (PKC) as well as the tyrosine kinase, Src. The activities of protein kinase A (PKA) and mitogen-activated protein kinase kinase (MEK) are speculated to contribute to GC-synchronisation (75). Liska *et al. *(75) reported that pharmacological inhibitors of PKA and MEK attenuate GC-mediated phase shifts. Whilst intriguing, this hypothesis warrants further investigation.

### Regulation of mRNA

Given the GR’s affinity for DNA, it is not surprising that the GR also directly regulates gene expression at the post-transcriptional level through RNA binding (76, 77, 78). Microarray analysis identified 479 GR-associated transcripts, enabling determination of a GR-binding mRNA structural hairpin loop motif (76). Using this motif, the authors derive thousands of putative GR mRNA targets, including *PER1* and *PER2* (76). *PER* mRNA binding could be a mechanism by which the GR regulates gene expression *post*-transcriptionally, perhaps through stabilisation of *PER* mRNA and/or promotion of its translation into protein. Alongside transcriptional induction of *PER1* and *PER2*, this potential post-transcriptional GR activity may expedite a rapid, pronounced increase in PER abundance, which, according to the TTFL model (Fig. 1A), would reset the phase of cellular rhythms. This hypothesis is further supported by ribosome profiling, which has identified 464 genes that are exclusively under translational regulation by the GR (80). Although the evidence for this is reasonably strong, the underlying mechanism is unclear at present.

Liška *et al.* have reported that DEX treatment of mouse choroid plexus explants leads to an acute induction in *de novo* PER2 protein synthesis, independently of a *PER2* RNA induction, as well as shortening the apparent half-life of PER2 protein (75). This apparent change in PER2 protein turnover is speculated to be the driving force for GC-synchronisation, constituting a rapid resetting signal.

## Critical problems with the canonical model for GC-synchronisation

Although the canonical model of GC-synchronisation outlined above conveniently marries together long-standing perceptions of GR signalling and cellular circadian timekeeping, there are substantial limitations of both the model and its supporting evidence.

### Circadian phase-resetting through *NR1D1* downregulation?

The hypothesis that GR-mediated downregulation of *NR1D1* resets the phase of circadian oscillations is not directly supported by experimental data – correlation does not demonstrate causation (Fig. 2). Within the TTFL framework, shifts in phase ultimately occur through changes in PER abundance and/or activity (15).

It is well established that GR activation leads to significant downregulation of *NR1D1* (45, 46); however, no evidence explicitly shows that this is capable of eliciting a circadian phase shift. It is true that BMAL1 is required for transcription of *PERIOD* and that REV-ERBa is one of several factors that regulate *BMAL1* transcription. However, the half-life of BMAL1 protein is ∼two days (82) and daily variation in abundance is only ∼10%. Given BMAL1’s long half-life and modest daily amplitude, it is unlikely to be limiting for *PER* transcription, so it is unclear whether acute GC-induced changes in *NR1D1* can alter PER expression via BMAL1.

### Transcriptional induction of *PER*?

Although GC-mediated transcriptional induction of *PER1* and *PER2* has been reported repeatedly (17, 26, 27, 42, 43, 75, 83), these studies all measured *PER* induction by quantifying total mRNA without discriminating between mature and nascently transcribed mRNA. It is well established that pools of mature mRNA can increase in abundance independently of an increase in nascent transcription by several mechanisms, for instance, an increase in processing of the nascent transcript pool, e.g. splicing, into mature RNA transcript (84), amongst others (83, 85, 86). Therefore, it is ambiguous whether the activated GR directly increases nascent* PER* transcription. More recent evidence described previously (75, 76, 80) raises the possibility that the GR regulates *PER*/PER at the post-transcriptional level, by either mRNA binding/stabilisation and/or direct regulation of protein synthesis/turnover.

A caveat to studies supporting GC-mediated *PER2* mRNA induction (26, 27) is their use of the potent synthetic GC, DEX, at concentrations at least 10X higher than physiologically equivalent levels of endogenous GCs (5). At these saturating concentrations, the GR is known to bind to lower-affinity genomic sites that the receptor would not otherwise occupy. Thus, whilst there is no doubt that *PER2* can be induced by GCs, it has not been demonstrated that this occurs *in vivo* in healthy humans or rodents to any meaningful extent. In fact, investigations using physiologically relevant concentrations of synthetic GCs revealed *PER1* as the only clock gene that shows an acute induction in mRNA expression (43, 75). Still absent, however, is functional validation that acute GC-mediated induction of *PER1* and/or *PER2* by the GR is necessary and sufficient for circadian resetting.

### GR–CLOCK protein interactions?

Several groups have proposed that the GR interacts directly with clock proteins, specifically BMAL1, CLOCK, REV-ERBa and CRY (32, 45, 54, 55). These interactions have either only been observed in an overexpression context or in the presence of unphysiological concentrations of the potent GC, DEX. Very high cellular concentrations of proteins resulting from overexpression are likely to instigate protein interactions, which would not otherwise occur physiologically (87). For TFs, in particular, overexpression can elicit well-established titration effects that are of uncertain relevance to endogenous function (88). Furthermore, there is inconsistency in the GR–clock protein interactions that have been reported. Caratti *et al.* demonstrated a direct interaction between the GR and CRY, and the GR and REV-ERB, but fail to replicate an interaction between the GR and CLOCK or BMAL1. Moreover, an experimental GR interactome study, which utilized BioID proximity labelling to generate a comprehensive list of cellular GR interactors, did not identify any of the clock proteins as significant interactors either in the presence or in the absence of GC ligand (89). Moreover, the differential subcellular localisation of REV-ERB and the GR is likely to occur in the presence of daily rhythms in endogenous GCs, but the evidence for reciprocal and antiphasic regulation is ambiguous. Therefore, whilst these clock proteins are certainly permissive for the expression of normal circadian transcriptional rhythms, it remains to be firmly established whether a direct functional interaction is required for circadian resetting.

### Reliance on pharmacological inhibitors

A further issue applying to many cellular studies of GC-synchronisation is a general reliance on pharmacological inhibitors to derive mechanistic underpinnings. Although pharmacological inhibitors are useful due to their rapid, reversible effects, there are several problems with using inhibitors as the sole basis for mechanistic claims. Many inhibitors lack specificity, for example, leading to off-target effects (90, 91), and can exhibit partial or inverse agonist activity (92).

For example, the claim that the GR is the exclusive mediator of GC-induced synchronisation (and not other receptors) is based on the use of the non-specific GR antagonist MIF (26, 27, 46). MIF is known also to bind to the progesterone receptor (PR) and androgen receptor (AR) and to exert partial agonist activity (28, 93). Current data therefore cannot rule out that GC-synchronisation acts partially through some of the other highly related nuclear hormone receptors.

Furthermore, inhibitors targeting major cellular pathways can elicit toxicity or stress responses that are epistatic to the phenotype under investigation, or affect it indirectly (94). For example, the use of the highly toxic actinomycin D by Torra *et al.* provides ambiguous support for the claim that downregulation of *NR1D*1 by GCs occurs at the transcriptional level. Similarly, the speculated role of the cytoplasmic kinases PKA and MEK is based on observations with the inhibitors H89 and U0126 (75), both of which have poor selectivity for the kinases in question with additional off-target effects on cellular metabolism and calcium homoeostasis (95, 96). At the high doses (10 and 25 μM, respectively) used by Liska *et al.*, it is difficult to be confident in a specific contribution by PKA and MEK to the GC-synchronisation mechanism.

### Caveats of KO systems

Finally, the proposed biological significance of CRY and REV-ERBa’s modulation of GR transcriptional activity is largely based on observations with CRY DKO and REV-ERBa KO mice, respectively (54, 55). A major limitation of gene deletion strategies is that the complete absence of a protein’s function often elicits multiple adaptations that perturb the underlying biology. Moreover, REV-ERBa and CRY proteins each have many functions that are unrelated to circadian regulation, which obscures any simple interpretation.

CRY DKO mice exhibit extremely disrupted circadian rhythms, which are highly variable and sensitive to external factors (97). In addition to effects on circadian physiology, these mice exhibit a range of pleiotropic physiological and behavioural changes due to CRY’s interaction with many signalling pathways as well as profound remodelling of the cellular proteome and phosphoproteome (98). CRY DKO mice also exhibit constitutively elevated circulating plasma GC levels and have elevated expression of cellular stress markers. Such factors may adequately account for many of the differences in GC signalling observed between CRY DKO and WT mice. In the same way, REV-ERBa KO mice have significant metabolic abnormalities (99), which disrupt the basal transcriptomic landscape, rendering it challenging to discern whether REV-ERBa has any direct interaction with GC transcriptional signalling.

## Concluding remarks on the GC-synchronisation mechanism

**Figure 3:**
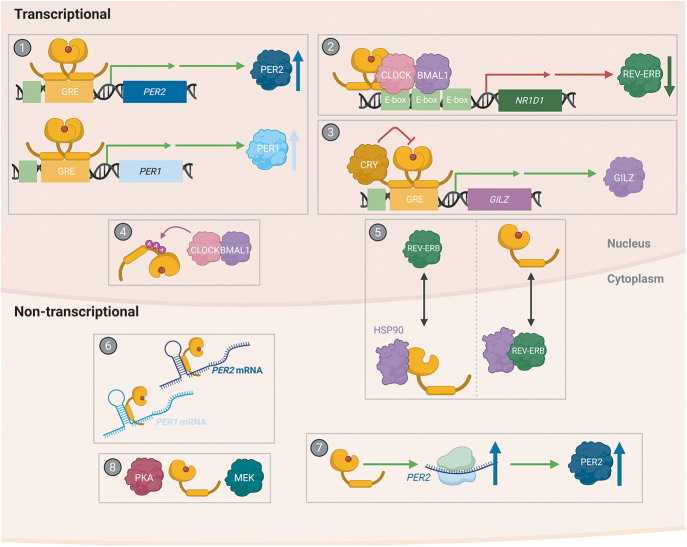
Transcriptional and non-transcriptional interactions between the GR and the circadian clock. Transcriptional interactions: i) GR-mediated transcriptional activation of *PER1* and *PER2* leading to an increase in PER1 and PER2 abundance (17, 26, 27, 41, 42, 43, 75). ii) Direct binding of the GR to CLOCK and BMAL1, which themselves bind to E-boxes upstream of *NR1D1*. Through tethering, the GR represses *NR1D1,* resulting in a decrease in REV-ERBa abundance (45, 46). iii) In a rhythmic fashion, CRY binds to the GR and represses the GR’s transcriptional activation activities (54). iv) CLOCK/BMAL1 acetylates the GR’s hinge region to suppress its transcriptional activities (32). Proposed non-transcriptional interactions: v) A reciprocal influence on nucleocytoplasmic localisation between REV-ERBa and the GR, both of which exhibit rhythmic localisation. This is proposed to occur through competition for HSP90 activity (38), i.e. REV-ERBa regulates GR activity depending on circadian phase (55). vi) Binding of the GR to a specific RNA hairpin loop motif, identified in *PER1* and *PER2* mRNA, to regulate stability and translation (76). vii) GR-mediated regulation of PER2 protein through an acute induction in *de novo* synthesis, independent of transcriptional activation (75). viii) The cytoplasmic kinases PKA and MEK contribute to GC-synchronisation (75). A full colour version of this figure is available at https://doi.org/10.1530/JOE-25-0340.

For the past few decades, research within both the circadian and GC signalling fields has focused its attention on transcription as the primary mediator of cellular timekeeping and GR signalling, respectively. However, it is now apparent that post-transcriptional mechanisms are important for regulation of both circadian rhythms (14, 100) and several critical GR signalling functions (60, 63, 64, 69, 70, 71). Several transcriptional and non-transcriptional interactions between the GR and the circadian clock have been proposed (Fig. 3). However, whilst some interactions are well supported by experimental evidence and very likely contribute to the mechanism of GC-synchronisation, the evidence supporting many of the other interactions seems spurious.

In light of current data, *PER1* mRNA/PER1 protein is the primary candidate for GC-synchronisation of cellular circadian rhythms in our view. GC induction of *PER1* mRNA and PER1 protein has been robustly observed and replicated in several independent studies. As discussed, an acute increase in PER protein abundance would lead to an increase in CRY/PER complex abundance, and thereby an increase in CLOCK/BMAL1 inhibition, regardless of the pre-existing phase of the core TTFL circuit (Fig. 2). Such an increase in CLOCK/BMAL1 inhibition should lead to phase shifts according to the widely accepted TTFL model. It is less clear, however, what determines the precise phase to which cells are reset nor why GC-mediated cues are more potent than other extracellular timing cues. Most importantly, the hypothesis that a *PER1*/PER1 induction is necessary and sufficient for GC-synchronisation of cellular circadian rhythms needs to be tested directly. Along these lines, we envisage several fruitful avenues for future investigation, as follows.

### Outstanding questions

- Which PER gene/protein paralogue is important for GC-synchronisation? Is there redundancy amongst paralogues?
- Is it an increase in nascent *PER* transcript and/or nascent PER protein that is required for circadian resetting to GCs?
- Is nascent* PER* transcription induced by physiologically relevant concentrations of GCs?
- Are other clock and clock-related genes, such as *NR1D1*, directly required or permissive (i.e. indirectly required) for GC-synchronisation?
- Does the GR signal non-canonically in the context of circadian synchronisation?
- If GC-synchronisation acts via the canonical model, what DNA-binding mode does the GR adopt? Which transcriptional co-regulators are important? Which PTMs of the GR are required?
- Is change in GR subcellular localisation important for synchronisation?
- Are there any tissue or cell-type specific differences in the mechanism of GC-synchronisation?
- How do GCs set cells to their precise phase, one which is 3–4 h earlier than other phase-resetting cues, such as insulin signalling? What kinetics govern this?
- How do GCs outcompete other phase-resetting cues?

## Declaration of interest

The authors declare that there is no conflict of interest that could be perceived as prejudicing the impartiality of the work reported.

## Funding

This work was supported by the Medical Research Council, as part of United Kingdom Research and Innovation (also known as UK Research and Innovation) MC_UP_1201/4. This study was also supported by the National Institute for Health and Care Research (NIHR) Oxford Health Biomedical Research Centre. The views expressed are those of the author(s) and not necessarily those of the NIHR or the Department of Health and Social Care. This work was supported by the NIHR Oxford Health Biomedical Research Centre, grant reference number: NIHR203316.
